# Maintenance of Noninvasive Brain Stimulation for Preventing Relapse in Depression: A Systematic Review and Meta-Analysis

**DOI:** 10.31083/AP49140

**Published:** 2025-12-16

**Authors:** Ru Wang, Xiumei Hou, Rui Li, Bochao Cheng, Cong Zhou, Chuang Xue, Kun Li, Wei Deng

**Affiliations:** ^1^Department of Child and Adolescent Psychiatry, Shandong Daizhuang Hospital, 272051 Jining, Shandong, China; ^2^Jining Key Laboratory of Neuromodulation, 272051 Jining, Shandong, China; ^3^Department of Radiology, West China Second University Hospital of Sichuan University, 610041 Chengdu, Sichuan, China; ^4^School of Mental Health, Jining Medical University, 272002 Jining, Shandong, China; ^5^Physical Integrated Diagnosis and Treatment Center, Affiliated Mental Health Center & Hangzhou Seventh People’s Hospital, Zhejiang University School of Medicine, 310058 Hangzhou, Zhejiang, China; ^6^Institute of Psychology, University of Chinese Academy of Sciences, 100101 Beijing, China; ^7^Liangzhu Laboratory, MOE Frontier Science Center for Brain Science and Brain-machine Integration, State Key Laboratory of Brain-machine Intelligence, Zhejiang University, 311121 Hangzhou, Zhejiang, China

**Keywords:** depressive disorder, electroconvulsive therapy, repetitive transcranial magnetic stimulation, transcranial direct current stimulation, relapse, maintenance treatment

## Abstract

**Background:**

Depression relapse rates remain high after acute treatment; this study evaluates the efficacy of maintenance noninvasive brain stimulation in preventing relapse and identifies optimal treatment parameters.

**Methods:**

This meta-analysis was conducted following PRISMA guidelines. We conducted a systematic search of PubMed, Embase, Web of Science, Cochrane Library, and PsycINFO databases up to January 5, 2025. The primary outcome was relapse rate.

**Results:**

A total of nine randomized controlled trials with 837 participants were included, six studies used electroconvulsive therapy (ECT) and three studies used repetitive transcranial magnetic stimulation (rTMS). Our findings indicate that ECT combined with pharmacotherapy or rTMS alone demonstrated superiority over pharmacotherapy alone in reducing the relapse of depression during 6, 9, 12-month maintenance treatment periods. Interestingly, ECT alone did not show significant results. In terms of stimulation parameters, the ECT combined with pharmacotherapy group mainly received right unilateral stimulation, while the ECT alone group had bitemporal stimulation. The stimulation frequency was similar between the two groups. In contrast, the rTMS-alone group had significantly higher stimulation frequencies than the ECT groups. We did not find any eligible studies on transcranial direct current stimulation, transcranial alternating current stimulation or magnetic seizure therapy, but they also showed potential in the maintenance treatment of depression, which warrants further investigation.

**Conclusions:**

ECT combined with pharmacotherapy, or rTMS alone, is more effective than pharmacotherapy alone in preventing relapse of depression during 6 to 12 months of maintenance treatment. Future research should prioritize identifying the optimal treatment regimen and exploring the potential of combination therapies.

**The PROSPERO Registration:**

CRD42023490546, https://www.crd.york.ac.uk/PROSPERO/view/CRD42023490546.

## Main Points

(1) Electroconvulsive therapy (ECT) plus pharmacotherapy or repetitive transcranial magnetic stimulation (rTMS) alone has shown significant efficacy.

(2) ECT alone did not show significant results.

(3) rTMS increases response rates, whether used alone or with pharmacotherapy.

## 1. Introduction

Global estimates indicate that over 350 million individuals are affected by depression, with projections suggesting it will become the largest global burden of disease by 2030 [1]. Depression is known for its high relapse rate and chronic course. After achieving remission with the initial treatment, approximately 50% of patients will experience a relapse, with the relapse rate increasing up to 80% after multiple episodes [2]. Pharmacotherapy is often the preferred treatment for depression; however, it may be associated with problems such as polypharmacy, adverse effects, or ineffectiveness, leading to treatment discontinuation [3, 4, 5]. Consequently, there is an urgent need for new therapeutic options.

Recently, noninvasive brain stimulation (NIBS) techniques, including electroconvulsive therapy (ECT), repetitive transcranial magnetic stimulation (rTMS), transcranial direct current stimulation (tDCS), transcranial alternating current stimulation (tACS) and magnetic seizure therapy (MST), have been increasingly used in the treatment of depression, demonstrating efficacy in the acute phase [6, 7]. However, the maintenance of treatment effects remains a challenge. Several studies indicate that maintenance NIBS regimens have the potential to prolong the effectiveness of acute treatment and reduce the risk of relapse in individuals with depressive disorders [8, 9, 10, 11, 12]. Elias *et al*. (2018) [8] found that maintenance ECT combined with pharmacotherapy was able to reduce depression relapse rate 1 year after the acute phase. Matsuda *et al*. (2023) [9] suggested that rTMS may reduce the risk of relapse in treatment-resistant depression (TRD) patients six months following acute treatment. Similarly, a separate study reported that rTMS could sustain mood stability in depressed patients for up to five months [10]. Additionally, Razza *et al*. (2021) [11] observed that individuals with major depressive episodes who received maintenance tDCS exhibited greater improvements in depression scores. These studies collectively highlight the benefits of maintenance NIBS in depressed patients. However, to date, no studies have directly compared the efficacy of different NIBS techniques in the maintenance treatment of depression. Therefore, our research aims to further integrate existing evidence on NIBS maintenance treatment modalities and compare the differences between several NIBS techniques.

## 2. Methods

This meta-analysis was conducted following the PRISMA guidelines (**Supplementary Table 1**) [13]. The review was registered with PROSPERO on December 16, 2023 under registration number CRD42023490546.

### 2.1 Search Strategies

Two authors independently searched articles published from the inception of the databases to January 5, 2025, in PubMed (https://pubmed.ncbi.nlm.nih.gov/), Embase (https://www.embase.com/), Web of Science (https://www.webofscience.com/), PsycINFO (https://psycnet.apa.org/databases/psycinfo), and the Cochrane Library (https://www.cochranelibrary.com/). The detailed search strategies are presented in **Supplementary Table 2**.

### 2.2 Eligibility Criteria

Studies were included according to the following Population, Intervention, Comparison, Outcome and Study (PICOS) framework [14]: (1) Participants: Patients met the diagnostic criteria for depression, including unipolar depression and bipolar depression [15, 16, 17]. (2) Interventions: Intervention involved one of the NIBS techniques (ECT, rTMS, tDCS, tACS, or MST) for a duration of 3 months or more. The duration of maintenance therapy for NIBS in depression is not standardized. Studies have reported durations ranging from 3 months [10, 11] to over 6 months [8, 9]. Consequently, we consider NIBS treatment lasting more than three months after acute treatment as maintenance therapy. (3) Comparison: Comparisons between NIBS with or without conventional treatment and other treatment methods. (4) Outcomes: Studies reporting relapse rate, response rate, remission rate, all-cause discontinuation rate, or depression scale scores. (5) Study design: Randomized controlled trails (RCTs).

#### Exclusion Criteria

Studies were excluded if they (1) included patients with a current diagnosis of schizophrenia or schizoaffective disorder; (2) had incomplete data in literature or failed to report required outcome indicators; (3) had inaccessible full texts; or (4) were animal studies, non-original research, or duplicate publications.

### 2.3 Data Extraction

The data extraction was carried out independently by two authors, and any disagreements were resolved through discussions or consultations with other investigators. For data that could not be obtained from the original studies, we referred to previous meta-analyses to extract relevant data. Where standard deviations were not available, they were calculated from standard errors, confidence interval (CI), *t*-values, or *p*-values. Additionally, attempts were made to obtain the missing data from the authors by email. Data that could not be obtained through these methods were excluded from the final analysis.

The primary focus of the study was to determine the relapse rate, while secondary outcomes encompassed response rate, remission rate, all-cause discontinuation rate, and depression scale scores. When available, data from intention-to-treat participants were prioritized over per-protocol participant data. Two studies had three arms: one study provided data from the ECT + Cognitive Behavior Therapy (CBT) group and the medication group [18], while the other study provide data on rTMS alone and medication therapy [19].

### 2.4 Risk of Bias Assessment

We employed the Cochrane tool and the modified Jadad scale to assess bias in randomized trials [20, 21]. The Cochrane tool includes six items, and each domain is scored as having a low, high, or unclear risk of bias at the study level. The modified Jadad scale consists of four items, with a total score of 7. Studies rated 4 or higher are considered to be of high quality. Disagreements between the two investigators were resolved through consensus, or consultation with other investigators.

### 2.5 Assessment of the Quality of the Evidence

We used the GRADEpro GDT (Version 15, McMaster University, Hamilton, ON, Canada) to assess the quality of evidence for each outcome based on five aspects, ultimately categorizing the evidence quality into four levels: high, moderate, low, and very low [22]. The assessment was conducted by two independent authors, and any disagreements that arose during the process were resolved by a third authors.

### 2.6 Statistical Analysis

The statistical analysis for this study utilized STATA/MP 17 software (StataCorp LLC, College Station, TX, USA) and Review Manager 5.3 (The Cochrane Collaboration, London, UK). Standardized mean difference (SMD) with 95% CI was used to express the continuous data, while risk ratio (RR) with 95% CI was used to calculate the categorical data. The Mantel-Haenszel χ^2^ test and *I*^2^ statistic for heterogeneity were conducted, with *I*^2^ > 50% indicating the existence of heterogeneity. When significant heterogeneity is present, we use subgroup analysis or meta-regression to explore the sources of heterogeneity. Additionally, we conducted sensitivity analysis using the leave-one-out method to test the robustness of the results. Due to the variability in intervention types across studies (e.g., stimulation frequency, brain region stimulated, NIBS technique, etc.), a common effect size could not be assumed, leading to the use of a random effects meta-analysis. Begg’s funnel plot and Egger’s test were used to investigate publication bias.

## 3. Results

### 3.1 Selection of Studies

**Fig. 1.**
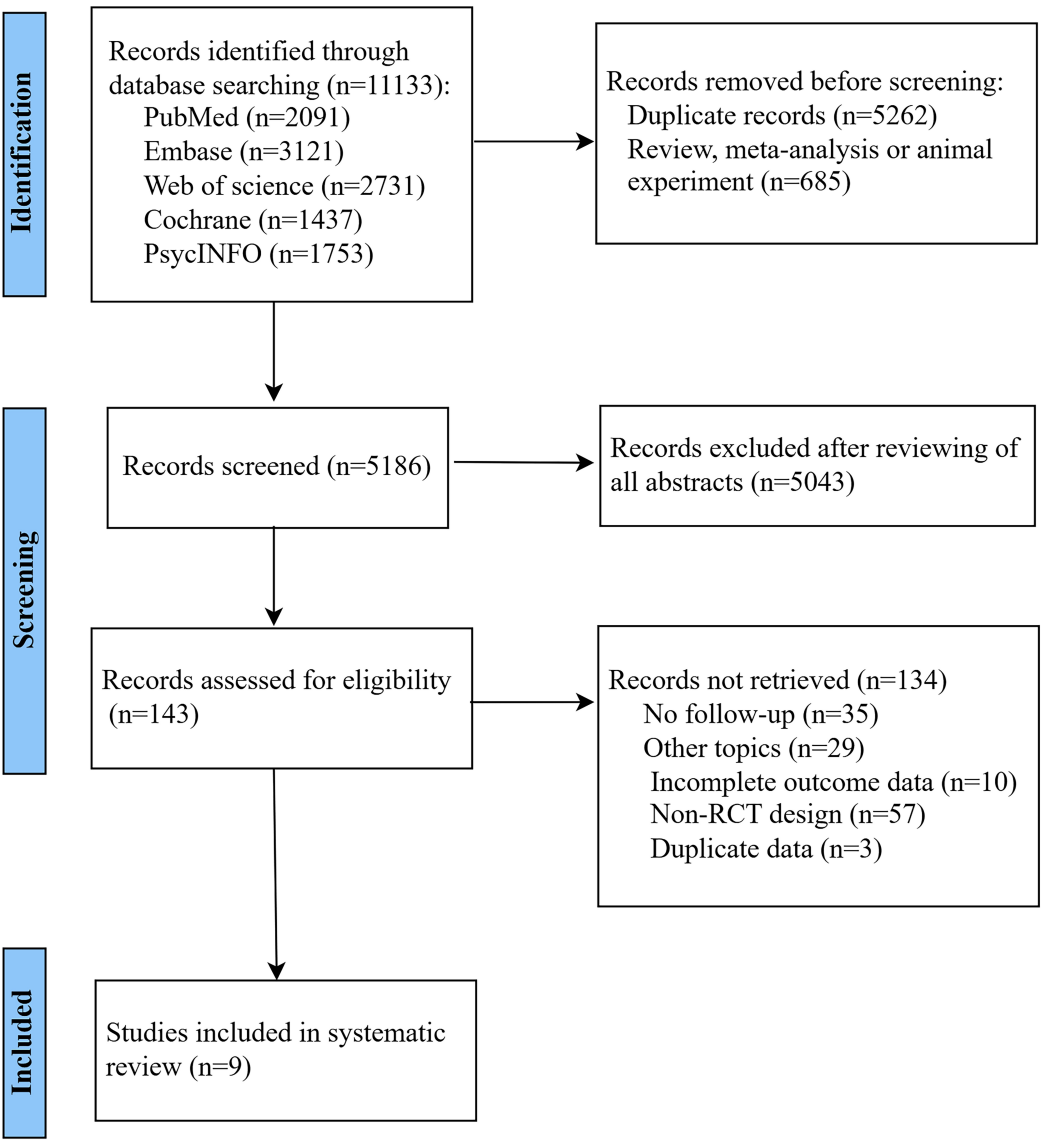
**Flowchart of study selection**.

The search identified 5186 abstracts, with 143 studies were considered for the full-text review (Fig. 1). After reading the full texts, the non-RCTs that did not match the purpose of this study, lacked outcome data, had no follow-up, or were repeated publications were excluded. Ultimately, 9 RCTs were included [18, 19, 23, 24, 25, 26, 27, 28, 29].

### 3.2 Study Characteristics

The characteristics of the RCTs included in the study are shown in Table 1 (Ref. [18, 19, 23, 24, 25, 26, 27, 28, 29]). A total of nine studies with 837 patients were included. Six studies used ECT for maintenance treatment [18, 23, 24, 25, 26, 29], while the remaining three studies used rTMS [19, 27, 28]. No eligible studies on tACS, tDCS and MST were found.

**Table 1.** **Basic characteristics of included studies**.

Author (year)	Groups	Mean age	Diagnosis	Acute phase	Criteria for maintenance therapy	Maintenance NIBS characteristics	Duration
Martínez-Amorós *et al*., 2021 [29]	ECT + Pharmacotherapy (n = 19) vs Pharmacotherapy (n = 18)	69 vs 67	Unipolar MDD	ECT	Remission	BT: weekly for 1 month, biweekly for 2 months, then monthly for 6 months (14 sessions).	9 months
Kellner *et al*., 2016 [26]	ECT + venlafaxine + lithium (n = 61) vs venlafaxine + lithium (n = 59)	70.8 vs 70.3	Unipolar MDD	RUL-UB ECT	Remission	RUL-UB: 4 ECT treatments in 1 month, then flexible treatments determined by STABLE algorithm.	6 months
Brakemeier *et al*., 2014 [18]	ECT + CBT + Pharmacotherapy (n = 25) vs Pharmacotherapy (n = 18) vs CBT + Pharmacotherapy (n = 17)	59.0 vs 62.4 vs 62.6	Unipolar MDD	RUL-UB ECT	Response	RUL-UB: weekly for 1 month, biweekly for 2 months, then monthly for 3 months (11 sessions).	6 months
Nordenskjöld *et al*., 2013 [25]	ECT + venlafaxine + lithium (n = 28) vs venlafaxine + lithium (n = 28)	52 vs 62	Unipolar/bipolar MDD	ECT	Response	RUL-UB: weekly for 6 weeks, biweekly for 46 weeks (29 sessions).	12 months
Navarro *et al*., 2008 [24]	ECT + nortriptyline (n = 16) vs nortriptyline (n = 17)	70.38 vs 70.65	Unipolar MDD	ECT + nortriptyline	Remission	BT: weekly for 1 month, then biweekly, finally monthly.	2 years
Kellner *et al*., 2006 [23]	ECT (n = 89) vs lithium + nortriptyline (n = 95)	59.6 vs 55.0	Unipolar MDD	ECT	Remission	BT: weekly for 1 month, biweekly for 2 months, then monthly for 2 months (10 sessions).	6 months
Benadhira *et al*., 2017 [28]	rTMS + Pharmacotherapy (10) vs Pharmacotherapy (7)	51.8 vs 58.1	Unipolar/bipolar TRD	rTMS	Response	Left DLPFC, 10 Hz, MT 110%: 3 sessions/week for 2 weeks, 2 sessions/week for 2 weeks, weekly for 2 months, biweekly for 8 months (34 sessions, 68,000 pulses).	11 months
Wang *et al*., 2017 [19]	Clustered rTMS (n = 91) vs Pharmacotherapy (n = 108) vs Clustered rTMS + Pharmacotherapy (n = 82)	42.3 vs 40.0 vs 40.9	Unipolar/bipolar TRD	antidepressants	Partial or full remission with acute antidepressants	Left DLPFC, 10 Hz, RMT 120% or 80%: 10 sessions over a 5-day period for 3 months and 5 sessions over a 3-day period for 9 months (34 sessions, 86,250 pulses).	12 months
Philip *et al*., 2016 [27]	rTMS (n = 23) vs Observation (n = 26)	48.2 vs 49.0	Unipolar TRD	rTMS	Response with acute rTMS	Left DLPFC, MT 120%: 1 session/four weeks for 12 months (12 sessions, 36,000 pulses).	12 months

Note: ECT, Electroconvulsive therapy; MDD, Major Depressive Disorder; BT, Bitemporal; RUL-UB, Right unilateral ultrabrief pulse; CBT, Cognitive Behavioral Therapy; rTMS, repeated Transcranial magnetic stimulation; DLPFC, Dorsolateral Prefrontal Cortex; MT, Motor threshold; RMT, Rest motor threshold; TRD, Treatment-Resistant Depression.

### 3.3 Quality Assessment of Included Studies

The Cochrane Collaboration’s tool for assessing risk of bias was used to evaluate the nine included RCTs [18, 19, 23, 24, 25, 26, 27, 28, 29], as shown in **Supplementary Figs. 1,2**. Six studies reported random sequence generation by computer methods [18, 19, 23, 24, 25, 26], but only two reported details about allocation concealment [25, 26]. A high risk of performance bias was identified in five studies, as both participants and assessors were unblinded [18, 23, 24, 25, 27]. Blinded outcome assessment was reported in only three trials [19, 23, 24]. Incomplete outcome data, indicating a high risk of attrition bias, were found in one study [26]. In terms of selective reporting bias, six studies were determined to have a low risk [19, 23, 24, 25, 26, 27]; and three had an unclear risk [18, 28, 29]. According to the modified Jadad scale, 80% of the included RCTs were rated as high quality [18, 19, 23, 24, 25, 26, 28] (**Supplementary Table 3**).

### 3.4 GRADE Evidence Quality

Ultimately, the quality of evidence for each outcome was rated as from very low to low. The GRADE of evidence was showed in **Supplementary Table 4**.

### 3.5 Outcomes

#### 3.5.1 Relapse Rate

Seven studies, including six utilizing ECT and one utilizing rTMS, were included in the analysis of relapse rate [18, 19, 23, 24, 25, 26, 29]. The control groups received pharmacotherapy. Data were collected at 6, 9 and 12 months of maintenance treatment.

**Fig. 2.**
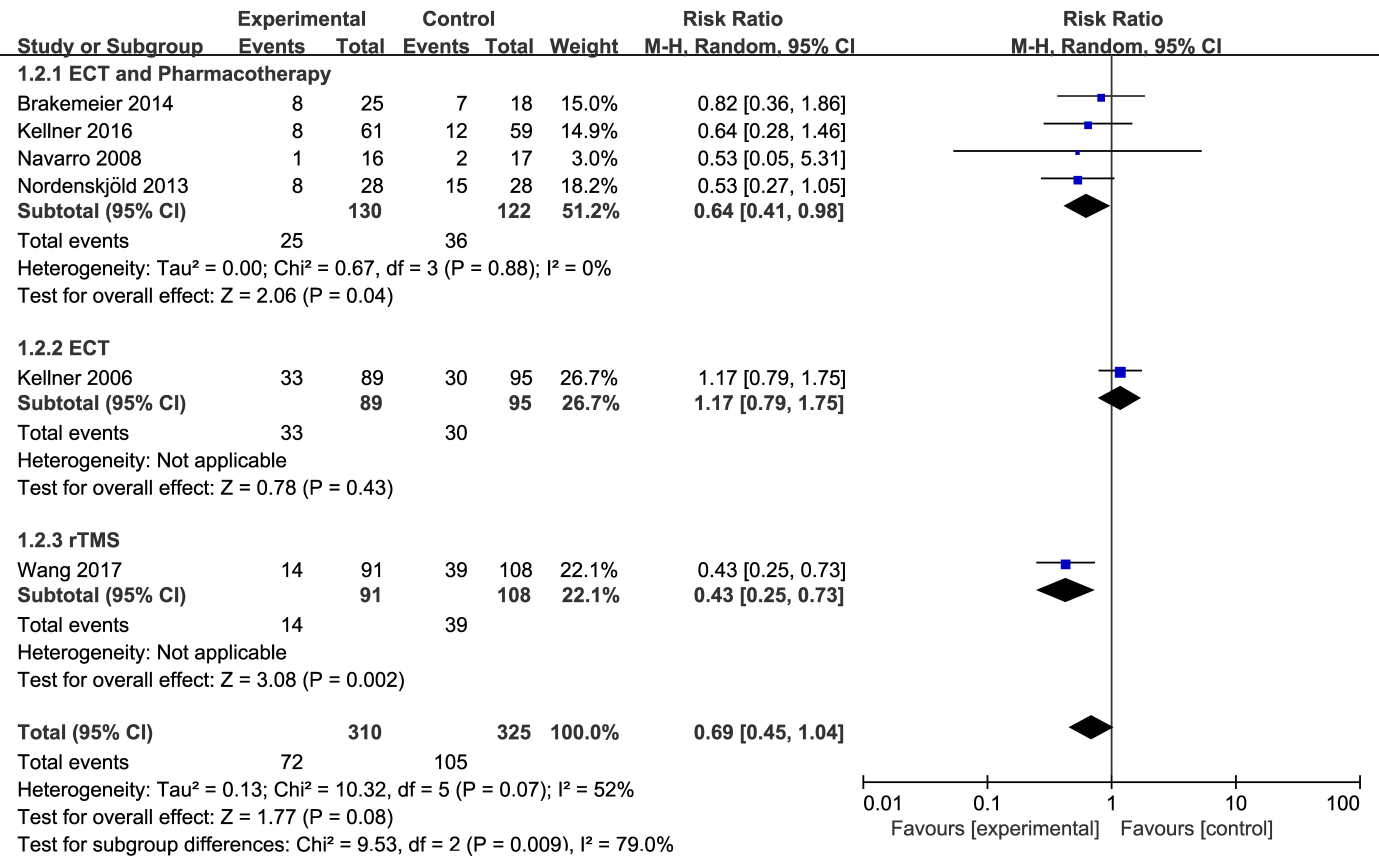
**Effect of maintenance NIBS with or without pharmacotherapy on relapse rate at 6 months**. Note: ECT, Electroconvulsive therapy; rTMS, repetitive Transcranial magnetic stimulation; NIBS, noninvasive brain stimulation.

The relapse rate at 6 months was reported in six studies [18, 19, 23, 24, 25, 26]. The meta-analysis, conducted using a random-effects model, revealed that maintenance NIBS did not significantly reduce the risk of depression relapse at 6 months (RR = 0.69, 95% CI: 0.45–1.04) (Fig. 2). The results of this sensitivity analysis revealed that when the study by Kellner *et al*., 2006 [23] was excluded, the overall effect became statistically significant (RR = 0.54, 95% CI: 0.39–0.76) (**Supplementary Fig. 3**).

No publication bias was detected for any outcome, as assessed by Begg’s funnel plot (**Supplementary Fig. 4**) and Egger’s test. Subgroup analysis based on the type of experimental group showed that ECT combined with pharmacotherapy (RR = 0.64, 95% CI: 0.41–0.98) and rTMS alone (RR = 0.43, 95% CI: 0.25–0.73) significantly reduced the relapse rate at 6 months, while ECT alone was ineffective (RR = 1.17, 95% CI: 0.79–1.75). Most studies in the ECT combined with pharmacotherapy group used right unilateral stimulation, while the ECT alone group received bitemporal stimulation. The two groups showed minimal differences in stimulation frequency.

**Fig. 3.**
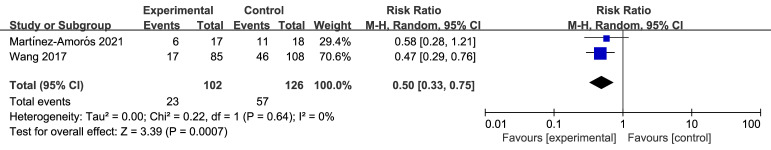
**Effect of maintenance NIBS with or without pharmacotherapy on relapse rate at 9 months**.

**Fig. 4.**
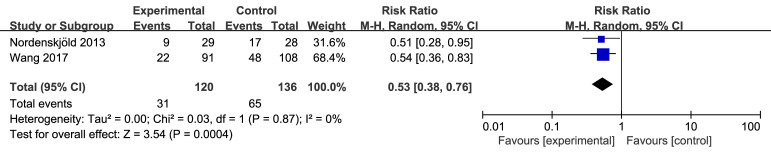
**Effect of maintenance NIBS with or without pharmacotherapy on relapse rate at 12 months**.

Data were also extracted at the 9-month [19, 29] and 12-month [19, 25] time points of maintenance therapy. The results indicated that ECT with pharmacotherapy or rTMS alone demonstrated efficacy in reducing depression relapse at both 9 months (RR = 0.50, 95% CI: 0.33–0.75) and 12 months (RR = 0.53, 95% CI: 0.38–0.76) as illustrated in Fig. 3 and Fig. 4 respectively.

#### 3.5.2 Response Rate

Response rate was reported in two studies [27, 28]. One study compared the effectiveness of rTMS in combination with pharmacotherapy versus pharmacotherapy alone, while the other study compared rTMS with an observational group. Data extracted at 6 months of maintenance treatment revealed a significant impact of rTMS with or without pharmacotherapy on response rates (RR = 2.33, 95% CI: 1.11–4.86) (**Supplementary Fig. 5**).

#### 3.5.3 Remission Rate

Three studies examined remission rates following 6 months of maintenance treatment [23, 27, 28]. No significant difference between the NIBS with or without pharmacotherapy and the control group (RR = 1.17, 95% CI: 0.72–1.90) (**Supplementary Fig. 6**). Subgroup analysis for either ECT alone (RR = 0.99, 95% CI: 0.73–1.36) and rTMS with or without pharmacotherapy (RR = 2.05, 95% CI: 0.77–5.43) showed no statistically significant differences.

#### 3.5.4 All-Cause Discontinuation Rate

All-cause discontinuation rate was reported in nine studies, with data extracted at 6 months and 12 months of maintenance treatment [18, 19, 23, 24, 25, 26, 27, 28, 29]. Meta-analysis did not find significant differences compared to the control group at either 6 months (RR = 0.78, 95% CI: 0.59–1.04) or 12 months (RR = 1.03, 95% CI: 0.52–2.05) (**Supplementary Figs. 7,8**).

#### 3.5.5 Depression Scale Score

Two studies, all using rTMS with or without pharmacotherapy, reported depression scores [27, 28]. Due to the use of different scale in original studies, SMD were used to pool effect sizes and increase comparability. Depression scores extracted at 6 months showed that rTMS with or without pharmacotherapy did not significantly reduce the patients’ depression scores (RR = –0.26, 95% CI: –0.75–0.23) (**Supplementary Fig. 9**).

#### 3.5.6 Adverse Effect

Two studies utilizing ECT reported Minimum Mental State Examination (MMSE) scores at 6 months [23, 25]. The meta-analysis revealed no statistically significant differences in MMSE scores between ECT (with or without pharmacotherapy) and pharmacotherapy alone (RR = –0.80, 95% CI: –3.55–1.95) (**Supplementary Fig. 10**). Other studies involving ECT, though not reporting specific scores, also confirmed no significant differences in MMSE scores between groups. Studies using rTMS reported mild and transient side effects, such as headache and sweating.

## 4. Discussion

This meta-analysis aims to clarify the long-term efficacy of maintenance non-invasive brain stimulation in preventing relapse of depression and its standardized clinical application. Our analysis of 9 studies involving 837 participants revealed that combining ECT with pharmacotherapy or using rTMS alone was more effective in decreasing depression relapse during the 6, 9, and 12-month maintenance treatment periods. In contrast, the utilization of maintenance ECT in isolation was ineffective, which is consistent with previous investigations on ECT efficacy [8]. Notably, rTMS with or without pharmacotherapy was associated with increase response rates at 6 months of maintenance treatment. However, maintenance NIBS with or without pharmacotherapy did not significantly enhance remission rates at 6 months. No statistically significant differences were found in the all-cause discontinuation rate between the experimental and control groups at either 6- or 12- month follow-up assessments. Finally, maintenance rTMS with or without pharmacotherapy did not effectively improve depression scores at 6 months.

Meta-analysis findings indicate that the combination of maintenance ECT with pharmacotherapy proves more effective in preventing relapse. This enhanced efficacy may be attributed to synergistic multi-target mechanisms: both interventions modulate gamma-aminobutyric acid and glutamatergic transmission and regulate the hypothalamic-pituitary-adrenal axis [30, 31, 32]. Additionally, they exhibit similarities in enhancing synaptic plasticity. ECT modulates intracellular signaling pathways, enhancing the signaling of brain-derived neurotrophic factor. Similarly, antidepressants can bind to neurotransmitter receptors, such as tyrosine kinase receptor 2, to enhance brain-derived neurotrophic factor signaling, which in turn facilitates synaptic plasticity [30, 33]. Furthermore, these therapies may influence monoaminergic systems by regulating serotonin and norepinephrine activity, thereby improving depressive symptoms [34, 35]. Notably, antidepressants can lower seizure thresholds (ST), leading to more effective seizures, which provides new insights into how adjunctive antidepressants can enhance the therapeutic effects of ECT. However, different antidepressants have differing effects on the ST [36, 37]. This reminds us that while considering the synergistic effects of ECT and antidepressants in reducing the relapse of depression, we should also carefully select adjunctive medications for ECT to optimize treatment outcomes. In addition, various ECT parameters (such as treatment intervals, electrode placement, etc.) can affect the ECT treatment outcomes. Therefore, optimizing these variables when using ECT alone may result in better outcomes. Studies have shown that treatment intervals longer than 2 months can lead to a decrease in effectiveness [8]. In this meta-analysis, which included six studies on maintenance ECT, the majority gradually reduced the frequency of treatment within 6 months, with the longest interval being 1 month [18, 23, 24, 29]. When using maintenance ECT alone, shortening stimulation frequency or adopting a more flexible and personalized scheme may be crucial in preventing relapse. Electrode placement is another crucial component of ECT administration. In this meta-analysis, three studies placed electrodes in the bitemporal (BT) while the other three used right unilateral [18, 25, 26]. During acute treatment processes, right unilateral ultrabrief pulse (RUL-UB) ECT demonstrates fewer cognitive side effects compared to BT, and high-dose efficacy (six times ST) is equivalent to BT [6, 38]. Another meta-analysis demonstrated that RUL-UB ECT combined with pharmacotherapy proves effective in maintenance treatment [8]. Consistent with previous studies, the RCTs included in this paper did not demonstrate long-term cognitive adverse effects related to maintenance ECT [39]. However, it is possible that the average age of the patients in this study was older, and their cognitive functioning was lower at baseline. We should examine cognitive performance among younger individuals. Furthermore, the lack of specificity and sensitivity of traditional assessment tools appears to be a major reason for the ongoing debate about long-term cognitive impairment caused by ECT. The majority of RCTs examined in this study relied on the Minimum Mental State Examination for evaluating cognitive performance, which may have limitations [40].

Our study provided evidence that maintenance rTMS reduces depression relapse rates and enhances response rates. Three studies on maintenance rTMS were included [19, 27, 28], of which two studies [27, 28] used standard rTMS, and one employed clustered rTMS [19]. Among the studies that used standard rTMS, a 12-month study found no benefit from monthly rTMS alone compared with control group [27]. However, another study showed that rTMS and pharmacotherapy combined yielded better results, but this antidepressant effect disappeared when the frequency of stimulation was reduced to fortnightly [28]. These findings suggest that combination therapy or a higher frequency of stimulation may be more effective. A study using clustered rTMS (monthly sessions of 5–10 sessions over 3–5 days) demonstrated that rTMS, as either monotherapy or combination therapy, prevents relapse of depression as well as improves treatment adherence [19]. This suggests clustered TMS may be more effective for treating depression in the long run. Additionally, studies have reported the use of deep transcranial magnetic stimulation (dTMS) in maintenance therapy [41]. dTMS is a new type of rTMS that differs from standard rTMS in that customized coils have a greater effective depth of stimulation [42]. An included study combined dTMS with pharmacotherapy (fortnightly for 4 weeks followed by weekly for 8weeks) and found that its effect on state of mind persisted over 12 months [41]. It is necessary to evaluate the effects of different maintenance rTMS techniques and determine the optimal treatment regimen in the future. Generally, rTMS maintenance treatment begins after a response or remission to acute treatment [8, 43]. However, it is worth noting that two studies did not adhere to this recommendation yet still reported positive outcomes [19, 41]. Recent research has indicated that a significant proportion of patients may not respond immediately to acute rTMS protocols, but may instead exhibit a delayed response. Even individuals who do not initially benefit from standard rTMS treatments may still experience symptom relief with extended treatment regimens. There is a strong correlation between the number of courses administered and the level of symptom relief [44, 45]. Another research has classified patients into persistent responders, non-responders, slow responders, and rapid responders, with only those classified as persistent responders demonstrating notable enhancement following extended rTMS therapy [46]. Future investigations should prioritize the identification of optimal candidates for ongoing maintenance treatment. Additionally, it is imperative to investigate the potential of utilizing rTMS as a maintenance treatment for patients who received pharmacotherapy during the acute phase.

Our meta-analysis did not find any eligible studies on tDCS, tACS and MST, but these techniques show great potential in the treatment of depression. Compared to other neuromodulation methods, tDCS is relatively cost-effective, portable, safe, and easy to use. Previous study has found that patients showed prolonged remission when receiving tDCS as maintenance therapy [11]. There are currently no standardized treatment parameters for tDCS in the maintenance treatment of depression. The anodal target site for stimulation is primarily the left dorsolateral prefrontal cortex. An electric current intensity of 2 mA is almost universally used, with electrode sizes ranging from 25 to 35 cm^2^, and the duration typically lasting 20 to 30 minutes [11]. Most studies applied maintenance tDCS once a week, which is more frequent than ECT and rTMS, with few maintenance treatments lasting longer than 6 months [11]. Increasing the number of treatments may affect adherence, making home-based tDCS a good option [47]. It has been shown that tDCS can be used at home to maintain depression treatment [48, 49]. Thus, home-based tDCS offers a promising alternative for long-term research on the effects of tDCS on depression. Additionally, high-definition tDCS (HD-tDCS), as a technical advancement over conventional tDCS, can precisely enhance the functionality of targeted brain regions. While the therapeutic effect of a single session of conventional tDCS lasts approximately 2 hours, HD-tDCS can extend this duration up to 6 hours. This improved approach may reduce the required frequency of maintenance sessions while enhancing patient compliance [50]. Some studies have indicated that tDCS can improve cognitive function [51, 52]. It also has the potential to ameliorate the decrease in prefrontal cerebral blood flow and metabolism induced by ECT [53]. Thus, tDCS maintenance may have great significance in preventing the relapse of depression and promoting cognitive functioning after acute ECT [54]. Future experimental studies are necessary to answer this question.

tACS uses sinusoidal waves to modulate abnormal brain oscillations. A meta-analysis comprising four RCTs has shown that tACS is superior to sham tACS in improving depressive symptoms. Three studies used 15 mA at 77.5 Hz for 20 days with positive results, while one study using 1–2 mA at 10/40 Hz for 5 days reported negative results. Current intensity and treatment duration may affect depression treatment efficacy [7]. Recent study has found that high-intensity tACS can act on the entire brain and stimulate deep brain nuclei. This therapeutic method is effective, has fewer side effects, and its effects can last at least 8 weeks after treatment [55]. A case report showed that a patient who responded well to acute treatment achieved remission after 12 weeks of weekly 1–2 mA tACS treatment, which confirms that extending treatment duration can improve patient outcomes [56]. Therefore, combining high-intensity tACS with maintenance therapy may help prevent depression relapse over a period of time, a hypothesis that warrants further research.

MST employs magnetic fields to induce a generalized seizure, offering potential advantages over ECT in terms of maintaining robust antidepressant efficacy and reducing adverse effects [57]. Due to concerns about cognitive safety, the frequency of seizure therapy is currently limited to 2 or 3 times per week, but MST’s enhanced focality and lower stimulation intensity may allow for more frequent sessions. One study found that approximately 60% of participants experienced sustained improvements in depressive symptoms without adverse cognitive effects. Future studies comparing continuation MST to ECT are essential [12].

Still, there are a few limitations in this study. First, the study examined the effect of NIBS in maintaining depression, but only included studies using ECT and rTMS. Specifically, this review included only one study on rTMS for reducing depression relapse during maintenance treatment. Thus, more research is needed to confirm this effect. Second, the significant heterogeneity of the study protocols makes it difficult to determine which factors most influence the effectiveness of maintenance therapy. Finally, most of the RCTs included in this meta-analysis have small sample sizes and are low-quality. In five studies, the lack of blinding for participants and assessors increases the risk of performance bias. This could have inflated the effect sizes, magnifying placebo effects or assessment biases. Therefore, we should interpret the results with caution.

## 5. Conclusions

In our study, ECT combined with pharmacotherapy or rTMS alone was more effective in reducing relapse at 6, 9 and 12 months of maintenance treatment. Future validation in larger, high-quality RCTs and a comparison of the advantages and disadvantages between different NIBS. Furthermore, we propose several avenues for future research. Firstly, to identify which pharmacotherapy can enhance the effectiveness of maintenance NIBS. Secondly, the determination of the optimal treatment regimen for maintenance NIBS. Additionally, the investigation of reliable biomarkers for identifying the ideal population for maintenance therapy. Finally, we should explore the combination of different treatment regimen to increase efficacy while reducing side effects.

## Availability of Data and Materials

The datasets used and analysed during the current study are available from the corresponding author on reasonable request.

## Supplementary Material

Supplementary material associated with this article can be found, in the online version, at https://doi.org/10.31083/AP49140.

## References

[1] GBD 2017 Disease and Injury Incidence and Prevalence Collaborators (2018). Global, regional, and national incidence, prevalence, and years lived with disability for 354 diseases and injuries for 195 countries and territories, 1990-2017: a systematic analysis for the Global Burden of Disease Study 2017. *Lancet (London, England)*.

[2] Ebmeier KP, Donaghey C, Steele JD (2006). Recent developments and current controversies in depression. *Lancet (London, England)*.

[3] Rush AJ, Trivedi MH, Wisniewski SR, Nierenberg AA, Stewart JW, Warden D (2006). Acute and longer-term outcomes in depressed outpatients requiring one or several treatment steps: a STAR*D report. *The American Journal of Psychiatry*.

[4] Kearns B, Cooper K, Orr M, Essat M, Hamilton J, Cantrell A (2022). The Incidence and Costs of Adverse Events Associated with Antidepressants: Results from a Systematic Review, Network Meta-Analysis and Multi-Country Economic Model. *Neuropsychiatric Disease and Treatment*.

[5] Kikuchi T, Suzuki T, Uchida H, Watanabe K, Mimura M (2013). Association between antidepressant side effects and functional impairment in patients with major depressive disorders. *Psychiatry Research*.

[6] Mutz J, Vipulananthan V, Carter B, Hurlemann R, Fu CHY, Young AH (2019). Comparative efficacy and acceptability of non-surgical brain stimulation for the acute treatment of major depressive episodes in adults: systematic review and network meta-analysis. *BMJ (Clinical Research Ed.)*.

[7] Zheng W, Cai DB, Nie S, Chen JH, Huang XB, Goerigk S (2023). Adjunctive transcranial alternating current stimulation for patients with major depressive disorder: A systematic review and meta-analysis. *Frontiers in Psychiatry*.

[8] Elias A, Phutane VH, Clarke S, Prudic J (2018). Electroconvulsive therapy in the continuation and maintenance treatment of depression: Systematic review and meta-analyses. *The Australian and New Zealand Journal of Psychiatry*.

[9] Matsuda Y, Sakuma K, Kishi T, Esaki K, Kito S, Shigeta M (2023). Repetitive transcranial magnetic stimulation for preventing relapse in antidepressant treatment-resistant depression: A systematic review and meta-analysis of randomized controlled trials. *Brain Stimulation*.

[10] Brian Chen YC, Chou PH, Tu YK, Brunoni AR, Su KP, Tseng PT (2023). Trajectory of changes in depressive symptoms after acute repetitive transcranial magnetic stimulation: A meta-analysis of follow-up effects. *Asian Journal of Psychiatry*.

[11] Razza LB, De Smet S, Moffa A, Sudbrack-Oliveira P, Vanderhasselt MA, Brunoni AR (2021). Follow-up effects of transcranial direct current stimulation (tDCS) for the major depressive episode: A systematic review and meta-analysis. *Psychiatry Research*.

[12] Tang VM, Blumberger DM, Throop A, McClintock SM, Voineskos D, Downar J (2021). Continuation Magnetic Seizure Therapy for Treatment-Resistant Unipolar or Bipolar Depression. *The Journal of Clinical Psychiatry*.

[13] Page MJ, McKenzie JE, Bossuyt PM, Boutron I, Hoffmann TC, Mulrow CD (2021). The PRISMA 2020 statement: an updated guideline for reporting systematic reviews. *BMJ (Clinical Research Ed.)*.

[14] Moher D, Liberati A, Tetzlaff J, Altman DG, PRISMA Group (2010). Preferred reporting items for systematic reviews and meta-analyses: the PRISMA statement. *International Journal of Surgery (London, England)*.

[15] Simon GE, Von Korff M (2006). Medical co-morbidity and validity of DSM-IV depression criteria. *Psychological Medicine*.

[16] Maske UE, Buttery AK, Beesdo-Baum K, Riedel-Heller S, Hapke U, Busch MA (2016). Prevalence and correlates of DSM-IV-TR major depressive disorder, self-reported diagnosed depression and current depressive symptoms among adults in Germany. *Journal of Affective Disorders*.

[17] Pedersen SH, Stage KB, Bertelsen A, Grinsted P, Kragh-Sørensen P, Sørensen T (2001). ICD-10 criteria for depression in general practice. *Journal of Affective Disorders*.

[18] Brakemeier EL, Merkl A, Wilbertz G, Quante A, Regen F, Bührsch N (2014). Cognitive-behavioral therapy as continuation treatment to sustain response after electroconvulsive therapy in depression: a randomized controlled trial. *Biological Psychiatry*.

[19] Wang HN, Wang XX, Zhang RG, Wang Y, Cai M, Zhang YH (2017). Clustered repetitive transcranial magnetic stimulation for the prevention of depressive relapse/recurrence: a randomized controlled trial. *Translational Psychiatry*.

[20] Higgins JPT, Altman DG, Gøtzsche PC, Jüni P, Moher D, Oxman AD (2011). The Cochrane Collaboration’s tool for assessing risk of bias in randomised trials. *BMJ (Clinical Research Ed.)*.

[21] Oremus M, Wolfson C, Perrault A, Demers L, Momoli F, Moride Y (2001). Interrater reliability of the modified Jadad quality scale for systematic reviews of Alzheimer’s disease drug trials. *Dementia and Geriatric Cognitive Disorders*.

[22] Guyatt G, Oxman AD, Kunz R, Brozek J, Alonso-Coello P, Rind D (2021). Corrigendum to GRADE guidelines 6. Rating the quality of evidence-imprecision. J Clin Epidemiol 2011;64:1283-1293. *Journal of Clinical Epidemiology*.

[23] Kellner CH, Knapp RG, Petrides G, Rummans TA, Husain MM, Rasmussen K (2006). Continuation electroconvulsive therapy vs pharmacotherapy for relapse prevention in major depression: a multisite study from the Consortium for Research in Electroconvulsive Therapy (CORE). *Archives of General Psychiatry*.

[24] Navarro V, Gastó C, Torres X, Masana G, Penadés R, Guarch J (2008). Continuation/maintenance treatment with nortriptyline versus combined nortriptyline and ECT in late-life psychotic depression: a two-year randomized study. *The American Journal of Geriatric Psychiatry: Official Journal of the American Association for Geriatric Psychiatry*.

[25] Nordenskjöld A, von Knorring L, Ljung T, Carlborg A, Brus O, Engström I (2013). Continuation electroconvulsive therapy with pharmacotherapy versus pharmacotherapy alone for prevention of relapse of depression: a randomized controlled trial. *The Journal of ECT*.

[26] Kellner CH, Husain MM, Knapp RG, McCall WV, Petrides G, Rudorfer MV (2016). A Novel Strategy for Continuation ECT in Geriatric Depression: Phase 2 of the PRIDE Study. *The American Journal of Psychiatry*.

[27] Philip NS, Dunner DL, Dowd SM, Aaronson ST, Brock DG, Carpenter LL (2016). Can Medication Free, Treatment-Resistant, Depressed Patients Who Initially Respond to TMS Be Maintained Off Medications? A Prospective, 12-Month Multisite Randomized Pilot Study. *Brain Stimulation*.

[28] Benadhira R, Thomas F, Bouaziz N, Braha S, Andrianisaina PSK, Isaac C (2017). A randomized, sham-controlled study of maintenance rTMS for treatment-resistant depression (TRD). *Psychiatry Research*.

[29] Martínez-Amorós E, Cardoner N, Gálvez V, de Arriba-Arnau A, Soria V, Palao DJ (2021). Can the Addition of Maintenance Electroconvulsive Therapy to Pharmacotherapy Improve Relapse Prevention in Severe Major Depressive Disorder? A Randomized Controlled Trial. *Brain Sciences*.

[30] Maffioletti E, Carvalho Silva R, Bortolomasi M, Baune BT, Gennarelli M, Minelli A (2021). Molecular Biomarkers of Electroconvulsive Therapy Effects and Clinical Response: Understanding the Present to Shape the Future. *Brain Sciences*.

[31] Sarawagi A, Soni ND, Patel AB (2021). Glutamate and GABA Homeostasis and Neurometabolism in Major Depressive Disorder. *Frontiers in Psychiatry*.

[32] Cui L, Li S, Wang S, Wu X, Liu Y, Yu W (2024). Major depressive disorder: hypothesis, mechanism, prevention and treatment. *Signal Transduction and Targeted Therapy*.

[33] Casarotto PC, Girych M, Fred SM, Kovaleva V, Moliner R, Enkavi G (2021). Antidepressant drugs act by directly binding to TRKB neurotrophin receptors. *Cell*.

[34] Berton O, Nestler EJ (2006). New approaches to antidepressant drug discovery: beyond monoamines. *Nature Reviews. Neuroscience*.

[35] Kritzer MD, Peterchev AV, Camprodon JA (2023). Electroconvulsive Therapy: Mechanisms of Action, Clinical Considerations, and Future Directions. *Harvard Review of Psychiatry*.

[36] Pluijms EM, Kamperman AM, Hoogendijk WJ, Birkenhäger TK, van den Broek WW (2021). Influence of an adjuvant antidepressant on the efficacy of electroconvulsive therapy: A systematic review and meta-analysis. *The Australian and New Zealand Journal of Psychiatry*.

[37] Sellevåg K, Bartz-Johannessen CA, Oedegaard KJ, Nordenskjöld A, Mohn C, Bjørke JS (2024). Unmasking patient diversity: Exploring cognitive and antidepressive effects of electroconvulsive therapy. *European Psychiatry: the Journal of the Association of European Psychiatrists*.

[38] Kolshus E, Jelovac A, McLoughlin DM (2017). Bitemporal v. high-dose right unilateral electroconvulsive therapy for depression: a systematic review and meta-analysis of randomized controlled trials. *Psychological Medicine*.

[39] Yoldi-Negrete M, Gill LN, Olivares S, Lauzière A, Désilets M, Tourjman SV (2022). The effect of continuation and maintenance electroconvulsive therapy on cognition: A systematic review of the literature and meta-analysis. *Journal of Affective Disorders*.

[40] Guo Q, Wang Y, Guo L, Li X, Ma X, He X (2024). Long-term cognitive effects of electroconvulsive therapy in major depressive disorder: A systematic review and meta-analysis. *Psychiatry Research*.

[41] Rapinesi C, Bersani FS, Kotzalidis GD, Imperatori C, Del Casale A, Di Pietro S (2015). Maintenance Deep Transcranial Magnetic Stimulation Sessions are Associated with Reduced Depressive Relapses in Patients with Unipolar or Bipolar Depression. *Frontiers in Neurology*.

[42] Levkovitz Y, Isserles M, Padberg F, Lisanby SH, Bystritsky A, Xia G (2015). Efficacy and safety of deep transcranial magnetic stimulation for major depression: a prospective multicenter randomized controlled trial. *World Psychiatry: Official Journal of the World Psychiatric Association (WPA)*.

[43] Rachid F (2018). Maintenance repetitive transcranial magnetic stimulation (rTMS) for relapse prevention in with depression: A review. *Psychiatry Research*.

[44] Razafsha M, Barbour T, Uribe S, Behforuzi H, Camprodon JA (2023). Extension of transcranial magnetic stimulation treatment for depression in non-responders: Results of a naturalistic study. *Journal of Psychiatric Research*.

[45] Hutton TM, Aaronson ST, Carpenter LL, Pages K, Krantz D, Lucas L (2023). Dosing transcranial magnetic stimulation in major depressive disorder: Relations between number of treatment sessions and effectiveness in a large patient registry. *Brain Stimulation*.

[46] Chen X, Blumberger DM, Downar J, Middleton VJ, Monira N, Bowman J (2024). Depressive symptom trajectories with prolonged rTMS treatment. *Brain Stimulation*.

[47] Kumpf U, Palm U, Eder J, Ezim H, Stadler M, Burkhardt G (2023). TDCS at home for depressive disorders: an updated systematic review and lessons learned from a prematurely terminated randomized controlled pilot study. *European Archives of Psychiatry and Clinical Neuroscience*.

[48] Alonzo A, Fong J, Ball N, Martin D, Chand N, Loo C (2019). Pilot trial of home-administered transcranial direct current stimulation for the treatment of depression. *Journal of Affective Disorders*.

[49] Le B, Alonzo A, Bull M, Kabourakis M, Martin D, Loo C (2022). A Clinical Case Series of Acute and Maintenance Home Administered Transcranial Direct Current Stimulation in Treatment-Resistant Depression. *The Journal of ECT*.

[50] Kuo HI, Bikson M, Datta A, Minhas P, Paulus W, Kuo MF (2013). Comparing cortical plasticity induced by conventional and high-definition 4 × 1 ring tDCS: a neurophysiological study. *Brain Stimulation*.

[51] Baeken C, Remue J, Vanderhasselt MA, Brunoni AR, De Witte S, Duprat R (2017). Increased left prefrontal brain perfusion after MRI compatible tDCS attenuates momentary ruminative self-referential thoughts. *Brain Stimulation*.

[52] Muccio M, Walton Masters L, Pilloni G, He P, Krupp L, Datta A (2022). Cerebral metabolic rate of oxygen (CMRO2) changes measured with simultaneous tDCS-MRI in healthy adults. *Brain Research*.

[53] Abbott CC, Gallegos P, Rediske N, Lemke NT, Quinn DK (2014). A review of longitudinal electroconvulsive therapy: neuroimaging investigations. *Journal of Geriatric Psychiatry and Neurology*.

[54] Hu R, Li J, Lu Y, Luo H, Zhang Y, Wang X (2024). The effect of transcranial direct current stimulation (tDCS) on cognitive function recovery in patients with depression following electroconvulsive therapy (ECT): protocol for a randomized controlled trial. *BMC Psychiatry*.

[55] Wang H, Wang K, Xue Q, Peng M, Yin L, Gu X (2022). Transcranial alternating current stimulation for treating depression: a randomized controlled trial. *Brain: A Journal of Neurology*.

[56] Riddle J, Rubinow DR, Frohlich F (2020). A case study of weekly tACS for the treatment of major depressive disorder. *Brain Stimulation*.

[57] Deng ZD, Luber B, McClintock SM, Weiner RD, Husain MM, Lisanby SH (2024). Clinical Outcomes of Magnetic Seizure Therapy vs Electroconvulsive Therapy for Major Depressive Episode: A Randomized Clinical Trial. *JAMA Psychiatry*.

